# Collective Influence of Multiple Spreaders Evaluated by Tracing Real Information Flow in Large-Scale Social Networks

**DOI:** 10.1038/srep36043

**Published:** 2016-10-26

**Authors:** Xian Teng, Sen Pei, Flaviano Morone, Hernán A. Makse

**Affiliations:** 1 Levich Institute and Physics Department, City College of New York, New York, NY 10031, USA; 2 Department of Environmental Health Sciences, Mailman School of Public Health, Columbia University, New York, NY 10032, USA

## Abstract

Identifying the most influential spreaders that maximize information flow is a central question in network theory. Recently, a scalable method called “Collective Influence (CI)” has been put forward through collective influence maximization. In contrast to heuristic methods evaluating nodes’ significance separately, CI method inspects the collective influence of multiple spreaders. Despite that CI applies to the influence maximization problem in percolation model, it is still important to examine its efficacy in realistic information spreading. Here, we examine real-world information flow in various social and scientific platforms including American Physical Society, Facebook, Twitter and LiveJournal. Since empirical data cannot be directly mapped to ideal multi-source spreading, we leverage the behavioral patterns of users extracted from data to construct “virtual” information spreading processes. Our results demonstrate that the set of spreaders selected by CI can induce larger scale of information propagation. Moreover, local measures as the number of connections or citations are not necessarily the deterministic factors of nodes’ importance in realistic information spreading. This result has significance for rankings scientists in scientific networks like the APS, where the commonly used number of citations can be a poor indicator of the collective influence of authors in the community.

Identification of the most influential nodes in social networks has broad applications in a variety of network dynamics^1^,^2^,^3^,^4^,^5^,^6^,^7^,^8^,^9^,^10^,^11^,^12^. For example, in viral marketing, advertising a small group of influential customers to adopt a new product can inexpensively trigger a large scale of further adoption^1^,^2^,^3^,^4^; in epidemics control, the immunization of structurally important persons can efficiently halt global epidemic outbreaks in contact networks^8^,^9^,^10^,^11^; and in biological systems like brain networks, some significant nodes are responsible for broadcasting information and therefore locating and protecting them are crucial for the whole information processing system^12^. Given its practical significance, the problem of finding the optimal set of influencers in a given network has attracted much attention in network science^13^,^14^,^15^.

For a long time, researchers have developed numerous heuristic measures as predictors of nodes’ importance in information spreading. Among the most frequently used topological properties are the number of connections (degree)^7^,^8^, betweenness^16^ and eigenvector centralities^17^, PageRank^18^, k-core^19^,^20^,^21^,^22^,^23^, etc. All of them are established in the non-interacting setting, where nodes’ significance is evaluated by taking them as isolated agents. As a result, these ad-hoc approaches, designed for finding single superspreaders, fail to provide the optimal solution for the general case of multiple influencers. To address this many-body issue, a rigorous theoretical framework based on collective influence (CI) theory has recently been presented^24^,^25^. With a broader notion of influence – collective influence, the CI method pursues the goal of maximizing the overall influence of multiple spreaders. Such explicit optimization objective enables CI to give the minimal set of spreaders.

Although CI exhibits good performance with scalability in the optimal percolation model, more validation work regarding its efficacy in real-world information spreading still needs to be done. Previously, the lack of real data of information diffusion has led to the mainstream adoption of artificial spreading models to simulate spreading dynamics. However, the over-simplified spreading models usually neglect such important factors as activity frequency^26^, connection strength and behavioral preferences, thus fail to reproduce some observed characteristics of real information spreading^27^. More importantly, different models may produce model-dependent contradictory results^22^. Therefore, it is necessary to evaluate CI’s performance empirically through realistic information diffusion before applying it to real-world applications like marketing and advertising.

Here, we address this problem by tracking and analyzing the real-world information flows in a wide range of social media: journals of American Physical Society (APS), an online social network Facebook.com (Facebook)^28^, a microblogging service Twitter.com (Twitter) and a blog website LiveJournal.com (LiveJournal). Rather than tracking the spreading range of single spreaders^22^, we intend to investigate the overall spreading range, i.e., the collective influence, of multiple spreaders. To achieve this, the most straightforward idea is to extract and examine the real instances of information diffusion that are triggered by multiple spreaders. Unfortunately, such ideal multi-source spreading instances in which spreaders send out the same piece of message at the same time rarely exist in reality. Even though we can find such instances, the initial spreaders are hardly the same as the set of nodes selected by CI or other heuristic strategies, making the comparison between those methods impossible.

To overcome the aforementioned difficulties, we construct “virtual” multi-source spreading processes by following users’ behavioral patterns in the data. In particular, under the assumption that users will maintain their personal preferences in spreading processes, we measure the strength of directed social ties shown in historical diffusion records to represent the influence strength of a user imposing on another. For a node under influence of several spreaders, the overall influence on it is defined as the highest influence strength. In this way, we are able to quantify the collective influence imposing on the entire network, corresponding to the collective spreading range of virtual processes initiated by any given set of seeds. Through comparisons with competing heuristic methods, including high degree (HD)^7^,^8^, adaptive high degree (HDA)^24^, PageRank (PR)^18^ and k-core^19^,^20^,^21^,^22^, we find that the set of spreaders selected by CI can exert larger collective influence on the population with the same number of initial seeds. This provides a direct empirical validation of CI’s good performance in real information spreading. In addition, some individual properties such as the number of connections and citations, which were previously regarded as reliable predictors of influence, are found to be invalid in the context of collective influence. This in turn reflects that it is the interplay between spreaders that determines the collective influence rather than individual features.

## Results

### Introduction of Datasets

In the following empirical study, four datasets are examined: the journals of American Physical Society (APS), an online social network Facebook.com (Facebook)^28^, a microblogging service Twitter.com (Twitter), and a blog website LiveJournal.com (LiveJournal). All datasets are available at kcore-analytics.com. During the period of data collection, people not only maintain social relations with their friends but also interact with others to spread and receive information. Certainly, there are diverse manifestations with respect to the social relation and interaction in distinct platforms. For instance, in the academic data of APS, authors show their social relations, i.e. coauthorship, through jointly publishing articles, and they reveal their interactions and information transmission by citing others’ papers. While in the online social media like Facebook, Twitter and LiveJournal, users reflect their social relations by becoming “cyber friends”, and they interact with each other by creating, receiving, and transmitting messages^29^. With the collection of such information, we can obtain the full network structure as well as the empirical information flows. Details about these data are explained as follows.

- The American Physical Society (APS) is the world’s largest organization of physicists. APS data contains the information of all the scientific papers published on APS journals until 2005, including Physical Review A, B, C, D, E and Physical Review Letters. From the author lists and references of scientific publications, we can obtain the information about collaborations and citations. In total, there are 299,996 articles and 230,521 authors in the data, along with 2,356,525 records of citations. We construct the underlying collaboration network according to their coauthorship. If two authors have published one article together, one undirected edge is built between them. Beyond that, we trace the information diffusion based on the reference flows. If a scientist *i* cites one paper written by *j*, then we can say that information spreads from *j* to *i*.
- Facebook is an online social networking service. In Facebook, each registered user maintains a friend list, which is a good representation of actual social relationships. Users can exchange messages, post status updates and photos, share videos, and browse the posts published by their friends. The Facebook data contains the friend lists and the entire records of wall posts from the New Orleans regional network, over a period of two years from September 26th, 2006 to January 22nd, 2009. This data contains 63,731 users and 838,092 wall posts in total. The social network is extracted from the friend lists. If user *j* is added into user *i*’s friend list (or *i* is in *j*’s friend list), we assume that they are friends so that we build an undirected edge between them. According to the wall posts, we can infer the information diffusion flows. If user *i* makes comments on user *j*’s page, we presume that *i* has gained information from *j* to motivate him/her to write comments.
- Twitter is a microblogging service that enables users to send and read short word-limited messages called “Tweets”. In the 2016 election year, Donald Trump, who is the presumptive nominee of the Republican Party for President of the United States, has become one of the most popular topics being discussed in Twitter. From February 10, 2016 to March 14, 2016, we collect approximately 670,000 Tweets that contain the key word “Donald Trump” or “Trump”. In the collection of Tweets, we extract four kinds of Tweets: mention, replies, retweet and quote. A mention is a Tweet that contains another user’s @username anywhere in the body of the Tweet. A reply is a response to another user’s Tweet that begins with the @username of the person you’re replying to. Replies are also considered as mentions. Besides, a retweet is a re-posting of someone else’s Tweet, in which such character RT@username appears at the beginning to indicate that users are re-posting others’ content. A quote is a special form of retweet that users can write their own comments when they are re-posting. We consider the mention (and also reply) relationship as a representative of strong social ties and use them to construct the network structure. Meanwhile, we use retweets (and quotes) to obtain information flows. If user *i* retweets a Tweet from user *j*, we assume information diffuses from user *j* to user *i*.
- LiveJournal is a blog-sharing website where users can maintain friend lists, keep a blog, journal or diary. Our data contains the friend lists for all users and their blog posts published from February 14th, 2010 to November 21st, 2011, which involves 9,573,127 users and 3,462,504 records of blog reference. Similar to Facebook, we depend on the friend list to build the underlying network topology. More importantly, LiveJournal users usually add URL links pointing to other relevant blogs when they refer them. As a result, we could use the URL reference to trace the information diffusion among users.

The originally constructed network is indicated by 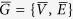 in which 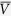 stands for the set of nodes and 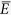 the set of edges. In the raw datasets of online social platforms including Facebook and LiveJournal, we find many inactive users who neither spread nor receive messages in network. Actually, they just register an account but do nothing during the period of time we collect data. Considering that no contributions are made by those inactive nodes to the information diffusion process, we exclude them from the original networks 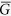 and construct an active network 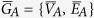. Different from the online social platforms, APS has no such inactive nodes as all the authors have to publish papers and cite others’ work. However, APS data contains a minority of articles (~0.67%) whose number of coauthors are so large (more than 100 coauthors) that they would produce extremely dense cliques. Therefore, we neglect those articles in constructing the APS network. In all the social networks, we only consider the largest connected component, denoted by *G* = {*V*, *E*}. Properties of the original and truncated networks are provided in Table 1.

### Construction of Virtual Information Spreading

In order to decide which strategy to use to locate the most influential nodes in networks, we intend to evaluate the collective influence exerted by the same number of influencers. The one that achieves the largest collective influence would be our first choice. To this end, the most straightforward idea is to compare the spreading range of multi-source spreading processes triggered by a fixed number of seeds selected by different methods. However, the multi-source spreading is an ideal process. In the ideal setting, multiple sources should be activated by the same piece of message at the same time. While in reality, such ideal situation rarely exists because of the intrinsic properties of real data. Users are interested in a wide range of topics, and they are receiving and delivering multifarious messages from time to time. It is unlikely that we can find enough real instances in which the spreaders happen to send out a same piece of message at the same time. Therefore, rather than enforcing real data to match the ideal expectation, we propose an alternative way - to construct a virtual multi-source spreading process.

**Figure 1:**
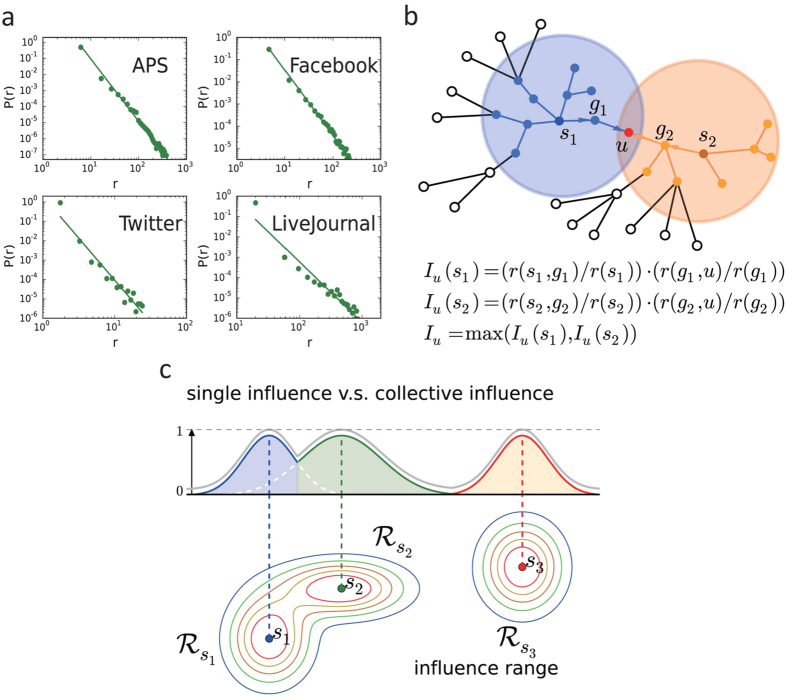
Construction of virtual spreading based on people’s interactions. (**a**) Distribution of directed tie strength for real networks. The power law distribution demonstrates the heterogeneity of interactions between nodes. (**b**) Calculation for influence strength. Nodes *s*_1_ and *s*_2_ are two distinct spreaders, the maximum spreading layer is set as *L* = 2. Node *u* is influenced by two seeds with the strength *I*_*u*_(*s*_1_) and *I*_*u*_(*s*_2_). We select the largest value to indicate the collective influence enforcing on it. (**c**) An illustration of single influence strength *I*_*u*_(*s*) along with collective influence strength *I*_*u*_. The three circle-like areas represent the corresponding influence ranges 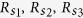 for distinct spreaders *s*_1_, *s*_2_, *s*_3_, and the contour lines indicate the levels of influence strength *I*_*u*_. When projecting it onto 2-dimensional space, we have the corresponding distribution. The collective outcome *I*_*u*_ (indicated by gray curve) is obtained by combining the single influence strengths of all the spreaders.

The main idea behind the virtual multi-source spreading processes is that users are expected to follow the behavioral patterns expressed in real data^30^. For user *i* with *k*_*i*_ neighbors who have chances to access information from *i*, the closely-tied neighbors interested in user *i*’s publications or posts would be more likely to inherit messages from *i*. On the contrary, those weakly-tied friends would occasionally be influenced by the information released from *i*. To reflect this effect, we propose a notion named the strength of directed ties *r*. For a directed link from *i* to *j*, the strength *r*(*i*, *j*) is defined by the number of messages, e.g., publications or posts, passed from *i* to *j*. By definition, the strength of directed tie *r*(*i*, *j*) from *i* to *j* is not generally equal to *r*(*j*, *i*) from *j* to *i*. Figure 1a reveals that the strength of directed tie follows a power-law distribution. We assume that, in the virtual processes, people would continue to maintain such behavioral patterns. In this way, we can approximate the multi-source information diffusion and obtain the collective influence as follows.

In the virtual processes, suppose a *q*-percentage of initial spreaders are activated at the beginning, denoted by *S* = {*s*_*i*_|*i* = 1, 2, ..., *n*, *n* = *N* ⋅ *q*}. We introduce a quantity *I*_*u*_(*s*) ∈ [0, 1] to represent the single influence strength that node *u* is affected by spreader *s*. Correspondingly, we employ *I*_*u*_ to indicate the collective influence strength enforced by all seeds *S*. Both of their calculations can rely on the above mentioned strength of directed ties (shown in Fig. 1b). For an arbitrary spreader *s*, the influence strength 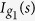 from *s* to its neighbor *g*_1_ depends on the strength of directed tie *r*(*s*, *g*_1_), or in other words, depends on the tendency of *g*_1_ to receive information from *s*. Assume that, during one period of time, *s* has totally sent out *r*(*s*) pieces of messages and *g*_1_ has accepted *r*(*s*, *g*_1_) of them [*r*(*s*, *g*_1_) ≤ *r*(*s*)]. The proportion of acceptance *r*(*s*, *g*_1_)/*r*(*s*) can be viewed as a proxy of influence strength from *s* to *g*_1_, i.e. 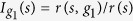. Next, *g*_1_ might affect its neighbor *g*_2_ ≠ *s* in the same way. Then we follow the spreading paths, multiply the proportions together and then acquire the influence strength *s* enforcing on its *l*-step neighbor *g*_*l*_, say


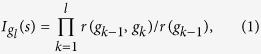


where *g*_0_ = *s*. Figure 1b gives an example with *l* = 2. As none of messages can spread infinitely, we set a number *L* as the maximum layer of spreading, so that the influence range, denoted by *R*_*s*_, could be approximated by a ball around *s* with the radius *L* (shown in Fig. 1c). Within each *R*_*s*_, we have 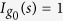 for the central spreader *s*, then the value decreases as *l* becoming larger, and 
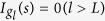
 for any external node. The schematic diagram regarding the distribution of influence strength within *R*_*s*_ can be seen in Fig. 1c. For APS and LiveJournal data, we know more information about references, the detailed calculation of influence strength is shown in Methods.

To obtain the collective influence *I*_*u*_ for node *u*, we apply


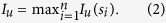


Referring to Fig. 1b,c, it is straightforward to understand when node *u* does not belong to any influence range, *I*_*u*_(*s*_*i*_) = 0 for any *i*, in which case the collective influence should be zero. For the case that node *u* is only influenced by one spreader, for example *I*_*u*_(*s*_*i*_) > 0 and *I*_*u*_(*s*_*j*_) = 0 for any *j* ≠ *i*, the collective influence should be chosen as the positive (largest) one *I*_*u*_ = *I*_*u*_(*s*_*i*_). More generally, if node *u* lies within the overlapping areas of more than one influence ranges, i.e. it is affected by more than one sources, we ought to choose the largest potential influence to be its collective influence during the virtual spreading process. Finally, we sum up all the {*I*_*u*_|*u* = 1, 2, ..., *N*} together to obtain the collective influence that spreaders impose on the entire system through


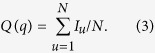


Since 0 ≤ *I*_*u*_ ≤ 1, we have 0 ≤ *Q*(*q*) ≤ 1, which corresponds to the collective spreading range for the virtual process (see Fig. 1c).

In general, the virtual process of multi-source spreading constructed here is an approximation of real information diffusion. We take advantage of real data to extract users’ behavioral patterns, base on which, we can calculate the single influence and collective influence that spreaders impose on each node. Given that, we can finally compute the collective influence exerted by all influencers on the entire network.

### Comparison of Different Methods

In this section, we compare CI algorithm with four other widely-used heuristic measures, including adaptive high-degree (HDA)^24^, high-degree(HD)^7^,^8^, PageRank (PR)^18^ and k-core^19^,^20^,^21^,^22^ (details about methods are shown in Methods). Recall that, our first step is to identify the *q*-percentage of initial spreaders according to different methods. Secondly, we construct a virtual multi-source spreading process. Finally, we compare the virtual spreading range *Q*(*q*), i.e. the collective influence of those initial influencers.

**Figure 2:**
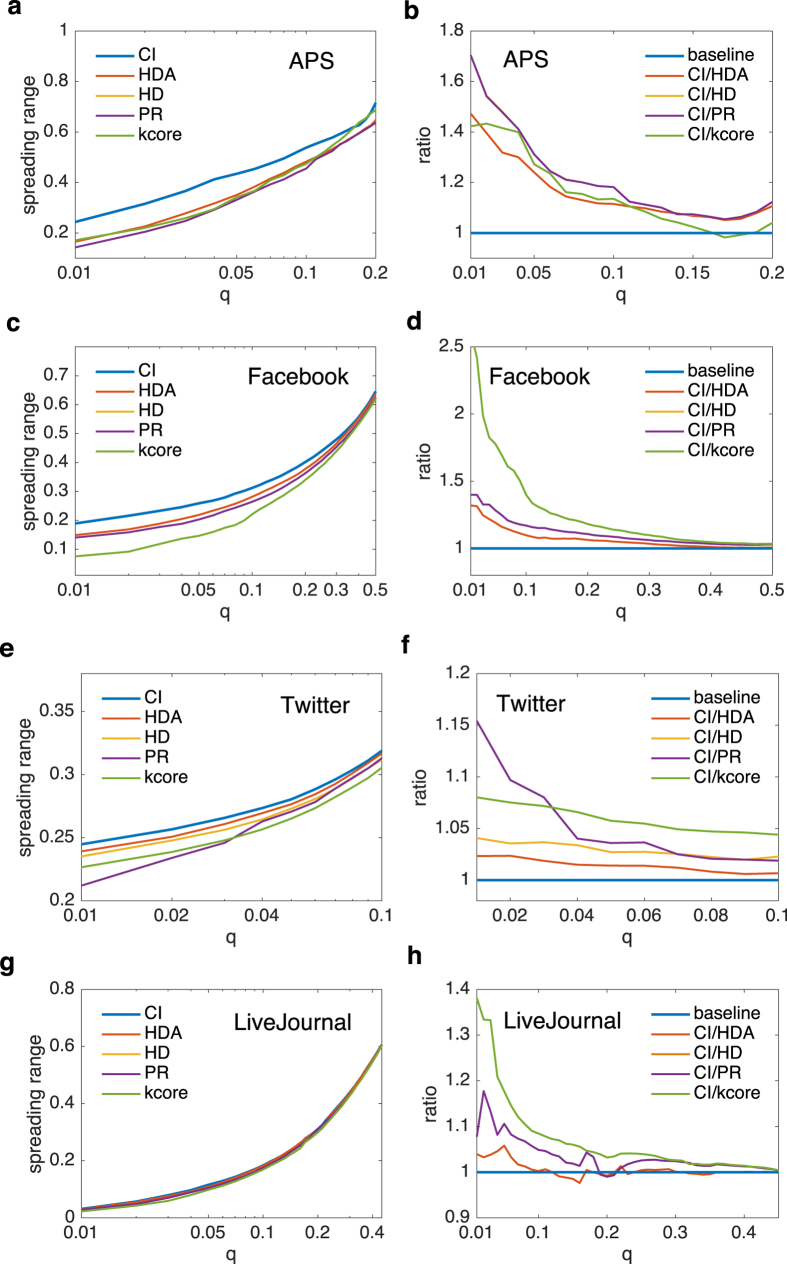
Performance of CI in large-scale real social networks. The datasets contain APS (**a,b**), Facebook (**c,d**), Twitter (**e,f**) and LiveJournal (**g,h**). We compare the virtual spreading ranges of different methods in (**a,c,e,f**). With a fixed fraction *q* of seeds, CI’s virtual spreading range is larger than all the heuristic approaches. Besides, we also show the ratios of spreading ranges between CI and others in (**b,d,f,h**). It reveals that the ratios are always larger than 1 (higher than the baseline), implying that CI is an effective strategy in locating multiple spreaders. We set *L* = 3 for APS and Facebook which have large value of 〈*k*^*d*^〉, and *L* = 5 for Twitter and LiveJournal that have small value of 〈*k*^*d*^〉. We care about the results when *q* is small, so we limit *q* within the range of small value. As *q* increases, the performances of all the strategies become similar.

Figure 2a,c,e,g show the virtual collective influence scores obtained by CI, HDA, HD, PR and k-core for the four networks – APS, Facebook, Twitter and LiveJournal. It can be seen that for a certain value of *q*, the set of nodes selected by CI can diffuse the information to a larger scale of populations than those obtained by other methods. CI’s good performance is more prominent for APS and Facebook data as their diffusion instances are relatively abundant. To clearly distinguish the performances of different methods, we also present the ratios between CI’s collective influence score and those of other approaches (Fig. 2b,d,f,h). It reveals that the ratios are always larger than one (indicated by the baseline at 1) for all datasets. Besides, the ratio is relatively large when *q* is small. As *q* increases, it would decline accordingly, suggesting that if we select a larger amount of influencers, the collective influence score obtained by all methods would become similar. Among the competing heuristic methods, HDA can be viewed as a special case of CI with the calculation radius being zero^24^ (see Methods). However, HDA’s capability in locating influencers is limited by the lack of knowledge of the surrounding nodes, so it is a strategy obtained from the non-interacting point of view. K-core method, a good predicator for locating single “superspreaders”^21^,^22^, whereas fails to identify multiple spreaders in the multi-source spreading process. This is because the selected influential nodes tend to cluster together in the core shells which induces large overlapping of their influence areas.

**Figure 3:**
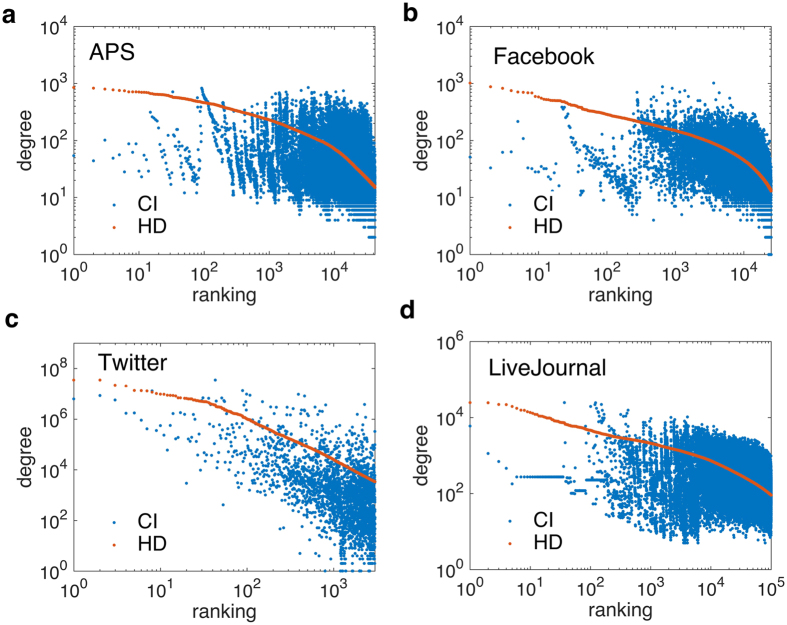
Degree versus ranking. We show the degrees of nodes ranked (from highest to lowest) by CI and HD for APS (**a**), Facebook (**b**), Twitter (**c**) and LiveJournal (**d**). It shows that CI can find those previously neglected weak nodes to emerge among most significant influencers. Meanwhile, some most connected nodes are ranked as moderate influencers by CI, indicating that such weak node effect is a consequence of collective influence in the case of multiple spreaders. This result has important consequences for ranking of researchers in scientific networks.

**Figure 4:**
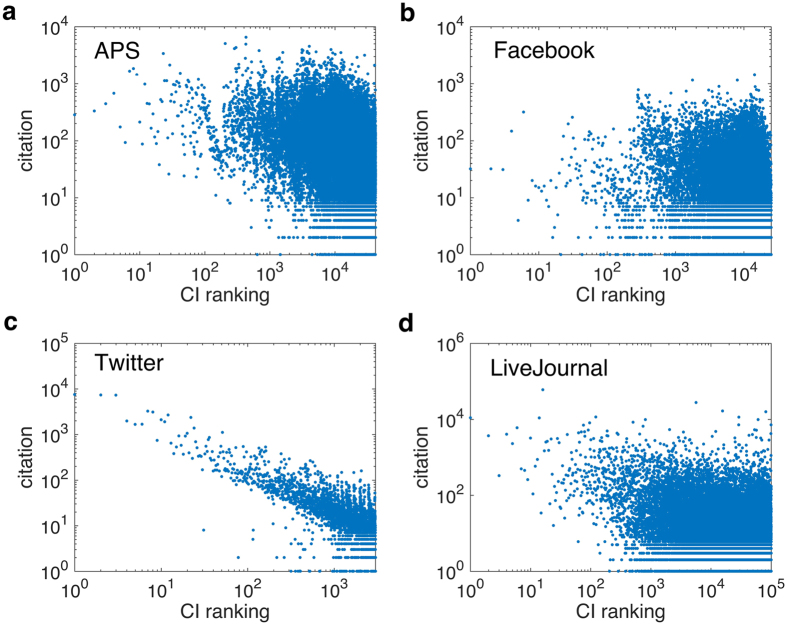
The number of citations versus CI ranking. We present the number of citations (comments, reposts or references) of nodes ranked by CI strategy for APS (**a**), Facebook (**b**), Twitter (**c**) and LiveJournal (**d**). Despite that in Twitter data, the most influential user is exactly the one with the largest amount of citations, the overall results still prove that large number of citations is not necessarily a reliable measure for identification of top-ranking influencers. This fact has meaning especially for academic rankings for physicists in community like APS. CI takes into account the maximization of influence in the whole network of each scientist rather than just the local information given by the number of citations. Thus a highly cited author may not have a large impact in the community if he/she is isolated in the periphery. An optimal measure as CI should rank such a scientist lower in the scientific community. This result calls for a revision of rankings based solely on the local information rather than the collective influence in the entire network community. We elaborate more on this problem in subsequent publications.

Besides, we also investigate the characteristics of influencers that CI has identified. Figure 3a shows the degree comparison of nodes ranked by CI and HD (from the most influential to the least). Unlike HD finding influencers just relying on degree, CI’s most important nodes contain not only hubs but also many weakly-connected nodes. Besides, some of the most connected nodes turn out to be moderate influencers. It confirms the former conclusion that collective influence is determined by the interplay of all the influencers. Under certain circumstances, some low-degree nodes surrounded by hierarchical coronas of hubs have larger contributions to collective influence than those high-degree nodes connecting to peripheral leaves^24^. In addition, we have also examined the correlation between CI ranking and the number of citations in Fig. 4. The number of citation for each user is defined as how many times other people have accepted or inherited information from him/her directly. We acquire such information through checking the citations (APS), comments (Facebook), retweets (Twitter) as well as URLs reference (LiveJournal). Except for Twitter, the other datasets show us that the most influential nodes are not necessarily those with the largest number of citations. The uniqueness of Twitter might be explained by considering the mechanism of network formation and the way of data collection. Twitter platform facilitates users arbitrarily following others, making it possible that super hubs with millions of followers emerge and hold significant influence; Besides, Twitter is gathered by focusing on a popular topic “Donald Trump”, the topic-based data might easily detect those extremely popular users who also play important role in spreading. Therefore, the phenomenon shown in APS, Facebook and LiveJournal suggest practical implications for academic rankings. When evaluating a researcher’s scientific impact within a field, his or her number of citation is not the determinative factor^31^,^32^. It also reminds us that influence is an emergent property arising from interactions rather than an evaluation by viewing nodes individually.

## Discussion

It is of importance to search for the most influential nodes in social networks. For a long time, heuristic approaches have been widely used to find superspreaders, yet without an ultimate solution for finding multiple influencers. Recently, a rigorous framework called collective influence (CI), along with a scalable algorithm, has been put forward to resolve the many-body problem. Even though CI has been shown to be effective in percolation model, we still need to verify its performance particularly in the real case of information diffusion. To achieve this, we collect data from four social media – APS journals, Facebook, Twitter as well as LiveJournal platforms. Different from the situation of finding single superspreaders where we check each node’s spreading range, under the circumstance of multiple spreaders, we should examine the collective spreading range. Given the difficulty that ideal multi-source spreading processes triggered by same messages at the same time are scarce in real-world diffusion, we propose a virtual multi-source spreading according to users’ behavioral patterns to approximate the ideal process. Finally, by comparing the collective influence, i.e. the spreading ranges in virtual process, we find that CI is effective in finding multiple influencers.

Moreover, our finding indicates that quantities from a non-interacting viewpoint, such as degree and the number of citations, are not reliable in measuring nodes’ importance in collective influence. Our investigation for influencers’ properties confirms that influence is an effect of cooperation in multi-source spreading. Our results can be transformed into an effective way to rank scientist in academic communities according to their collective influence rather than on the commonly used local connectivity metric, like the number of citations or collaborations in the H-index (Hirsch number). Using the number of citations, as shown in Fig. 4, can be a poor indicator of the collective influence of a researcher on other researchers in the community. A global quantity like the Collective Influence that takes into account the optimization of influence of all researchers at once, provides a meaningful ranking of researchers according to the maximization of their influence. More studies will follow to elaborate on this particular point.

## Methods

### Collective Influence Method

#### Collective Influence (CI) Algorithm

CI is an optimization algorithm that aims to find the minimal set of nodes that could fragment the network in optimal percolation^24^. In percolation theory^33^, if we remove nodes randomly, the network would undergo a structural collapse at a critical fraction where the probability that the giant connected component exists is *G* = 0. The optimal percolation is an optimization problem which attempts to find the minimal fraction of influencers *q*_*c*_ to achieve the result *G*(*q*_*c*_) = 0. Let the vector **n** = (*n*_1_, *n*_2_, ..., *n*_*N*_) represent whether a node is removed (*n*_*i*_ = 0) or not (*n*_*i*_ = 1), and the vector **v** = (*v*_1_, *v*_2_, ..., *v*_*N*_) represent whether a node belongs to the giant connected component (*v*_*i*_ = 1) or not (*v*_*i*_ = 0). The relationship between **n** and **v** can be derived in locally tree-like networks using message passing (MP) approach^34^,^35^:


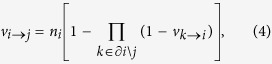


where *v*_*i*→*j*_ indicates the probability of *i* being in the giant component when *j* is absent, and ∂*i*\*j* is the neighbors of *i* besides *j*. The equation’s possible solution *v*_*i*→*j*_ = 0 for all *i* → *j* is associated with the special situation where the giant connected component is absent; therefore, to obtain *G*(*q*) = 0, the stability of this solution must be guaranteed. As a matter of fact, the stability of *v*_*i*→*j*_ = 0 is controlled by the largest eigenvalue *λ*(**n**; *q*) of the linear operator 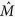, which is defined on the directed edges of networks as


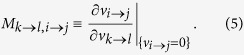


It can be expressed as


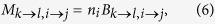


where *B*_*k*→*l*,*i*→*j*_ is the non-backtracking matrix of the network^36^,^37^. *B* stores the topological interconnections of network whose element *B*_*k*→*l*,*i*→*j*_ = 1 if *l* = *i*, *j* ≠ *k*. So far, the original optimal percolation problem has been rephrased as a mathematical statement: finding the optimal configuration of n* with size *q*_*c*_ that achieves the critical threshold:


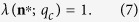


The eigenvalue *λ*(**n**; *q*) can be calculated according to power method^38^:


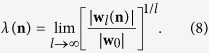


At a finite *l*, |w_*l*_(**n**)|^2^ is the cost energy function of influence that needs to be minimized. Take Equation 8 as a starting point, the problem of finding the optimal set of influencers can be solved by minimizing the following cost function:


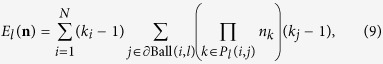


where Ball(*i*, *l*) is the set of nodes inside the ball of radius *i* around the central node *i*, and *P*_*l*_(*i*, *j*) is the shortest path of length *l* connecting *i* and *j*. To minimize the energy function of a many-body system, an adaptive method is developed with the main idea of removing the nodes causing the biggest drop in the energy function - CI algorithm. In general, CI algorithm can be stated as follows. Firstly, it considers the nodes at the frontier *j* ∈ ∂Ball(*i*, *l*) and assigns to node *i* a collective influence value at the level of *l* as


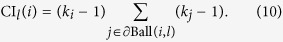


Starting with the node with the highest CI_*l*_, CI adaptively removes nodes and after each removal, it recalculates CI_*l*_ for all the rest nodes in the system. From the calculation we know that CI has richer topological contents and its performance will be improved as *l* increases, but no larger than the network diameter because this case amounts to random identification. In our analysis, we adopt the parameter *l* = 3 in the adaptive calculation of CI, which has been shown to be sufficient for optimal percolation. At the opposite extreme *l* = 0, we have CI_0_(*i*) = (*k*_*i*_ − 1)^2^. Under this situation, CI algorithm is reduced to the High-degree adaptive (HDA) method. For *l* ≥ 1, CI also considers the surrounding neighborhoods and the interactions among nodes; meanwhile, it is an easily-implemented algorithm as it only needs local topological structure within the ball of the radius *l* instead of the whole network structure. More importantly, its computational complexity is *O*(*N*log*N*), which guarantees its application for large real networks^39^.

### Heuristic Methods

#### k-core

In k-core method, nodes are ranked based on their *k*_*S*_ values, which are calculated during the process of *k*-shell decomposition^19^,^20^,^21^,^22^. In *k*-shell decomposition, nodes are removed iteratively. Firstly, nodes with *k* = 1 are removed and continue pruning the networks until no leaf nodes are available. The set of removed nodes compose the peripheral k-shell with index *k*_*S*_ = 1. Similarly, the next k-shells with index *k*_*S*_ > 1 are generated and the nodes located within the core area have the highest *k*_*S*_ values. Actually, in *k*-shell composition, all the nodes are divided into different shells according to their relative locations in networks. Compared with the peripheral nodes, the core nodes have higher probabilities to cause large-scale diffusions. This method has been revealed to perform well in searching for single spreaders who can yield large influence areas. However, it has a poor performance when being used to optimizing the collective spreading caused by multiple spreaders^21^. Because k-core would select a bunch of nodes within or near the network core, so their influence areas would heavily overlap and produce a bad collective outcome^21^.

#### PageRank(PR)

PageRank algorithm was firstly proposed by S. Brin and L. Page and used by Google in order to rank websites^18^. It extends the idea in academic citation that the number of citations or backlinks give some approximation of a page’s importance, by not counting links equally but normalizing by the number of links on a page. Its calculation is as follows: if page *A* has pages *T*_1_, ..., *T*_*N*_ citations with the associated PageRank as PR(*T*_1_), ..., PR(*T*_*N*_), then the PageRank of *A* is given by


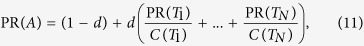


in which *C*(*A*) is defined as the number of links going out of page *A*. PageRank outputs a probability distribution used to represent the likelihood that a person randomly clicking on links will arrive at any particular page. The higher the probability, the higher the PR value of this page. In practice, PageRank can be calculated using a simple iterative algorithm and corresponding to the principal eigenvector of the normalized link matrix of the web network.

#### High-Degree(HD)

HD method ranks nodes directly according to the number of connections^7^,^8^. Compared with other methods requiring global network structures like k-core and PageRank, HD only needs local information and is easily implemented. However, it cannot deal with the circumstance in which hubs form tight community such that their spreading areas would heavily overlap^40^,^41^.

#### High-Degree Adaptive(HDA)

HDA is the refined adaptive version of HD method. To help mitigate the above mentioned situation, HDA recalculates the degrees after each removal. It can also be viewed as a special case of CI algorithm at *l* = 0. Compared with CI, HDA represents the one-body scenario where the influencers are considered in isolation and therefore, it lacks the collective influence effects from the neighborhood.

### Data Processing

#### Analyzing APS and LiveJournal

In terms of APS, we know the specific article pairs (*α*, *β*), which means paper *α* cites paper *β*, in other words, the authors *A*_*β*_ of *β* spread their scientific discoveries to the authors *A*_*α*_ of *α*. Therefore, for an arbitrary author *s*, we can know his or her journal set *J*(*s*) = {*J*_*i*_|*i* = 1, 2, ..., *n*_*s*_} in which *J*_*i*_ indicates each piece of paper and *n*_*s*_ stands for the number of papers published by *s*. By tracking the spreading for each paper *J*_*i*_ through citation flows, we can determine its influence range *R*_*s*_(*J*_*i*_) containing all people who have cited this paper *J*_*i*_. For each receiver *u* ∈ *R*_*s*_ = {*R*_*s*_(*J*_*i*_)|*i* = 1, 2, ..., *n*_*s*_}, we calculate the individual influence strength by 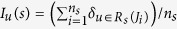 where 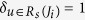 if and only if *u* ∈ *R*_*s*_(*J*_*i*_). Large values of *I*_*u*_(*s*) means that *u* is more likely to cite the work of *s* than other peers. Next, the collective influence strength from all sources can be obtained by 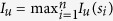. In LiveJournal, we know information about blog references. So, we can follow the similar method as in APS to process LiveJournal data.

## Additional Information

**How to cite this article**: Teng, X. *et al*. Collective Influence of Multiple Spreaders Evaluated by Tracing Real Information Flow in Large-Scale Social Networks. *Sci. Rep.* **6**, 36043; doi: 10.1038/srep36043 (2016).

**Publisher’s note:** Springer Nature remains neutral with regard to jurisdictional claims in published maps and institutional affiliations.

## Figures and Tables

**Table 1:** Properties of the original and processed networks 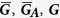 in this article.

Networks	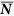	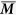	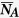	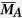	*N*	*M*	〈*k*〉	〈*k*^*d*^〉	*q*_*c*_
APS	230,521	1,607,305	230,521	1,607,305	190,161	1,582,710	16.4	37.4	20%
Facebook	63,731	817,090	45,746	703,924	45,459	703,803	31.0	18.8	45%
Twitter	311,334	151,654	311,334	151,654	29,463	143,220	9.7	5.1	6%
LiveJournal	9,573,126	188,240,039	304,858	19,785,460	290,362	19,783,730	136.3	7.7	46%

In the table, 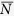 (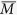) is the number of nodes (edges) in the original networks 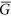, 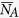 (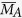) represents the number of nodes (edges) in the active network 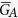, *N* (*M*) indicates the number of nodes (edges) in the network *G*. 〈*k*〉 is the average degree of network *G*. 〈*k*^*d*^〉 denotes the average out-degree of diffusion graph, i.e. the average number of messages which have been sent out. Besides, *q*_*c*_ indicates CI’s minimal fraction of influencers to fragment the networks in optimal percolation^24^. All datasets are available at kcore-analytics.com.
